# Splenic Solitary Metastasis From Melanoma Malignum: A Case Report

**DOI:** 10.7759/cureus.91038

**Published:** 2025-08-26

**Authors:** Mirela Piric

**Affiliations:** 1 General Surgery, Special Hospital "Medical Institute Bayer", Tuzla, BIH

**Keywords:** melanoma, metastasis, solitary metastasis, splenectomy, splenic neoplasms

## Abstract

Melanoma is a malignant tumor derived from melanocytes, predominantly located in the skin and mucosal surfaces. Although this cancer can spread to multiple organs, isolated metastasis to the spleen is exceptionally uncommon. When splenic involvement does occur, it typically forms part of widespread systemic dissemination, rather than appearing as a solitary lesion.

This report discusses a 62-year-old male who underwent elective splenectomy following the discovery of a suspicious splenic mass. Three years earlier, he underwent excision of a pigmented nodular lesion in the right pectoral region. Histopathology revealed superficial spreading melanoma, Clark III, Breslow II, with 0.8 mm margins and no lymphovascular invasion, microsatellitosis, or mitoses. Re-excision and right axillary dissection confirmed metastatic melanoma in one of eight lymph nodes. Examination of the resection margins of the re-excised specimen showed no evidence of increased melanocytic activity. A follow-up abdominal CT identified a 7 mm, well-circumscribed, hypodense lesion in the inferior pole of the spleen, which lacked contrast enhancement. Due to inconclusive findings on both CT and ultrasound, surgical removal of the spleen was performed. Subsequent histological and immunohistochemical evaluation confirmed the lesion to be metastatic amelanotic melanoma (ICD-O code M8770/6).

Solitary metastasis to the spleen from melanoma remains a clinical rarity and often manifests several years after the initial diagnosis. In such cases, surgical excision is considered the preferred treatment, offering potential for extended survival in the absence of additional metastatic disease.

## Introduction

Melanoma is a malignant tumor that arises from melanocytes, the pigment-producing cells of the skin [1]. As reported by the Surveillance, Epidemiology, and End Results (SEER) program of the National Cancer Institute, it currently ranks as the fifth most frequently diagnosed cancer among both male and female populations [2]. After the primary tumor develops, metastases most commonly affect the skin and subcutaneous tissues, followed by the lungs, liver, bones, and brain [3]. The time interval between the initial diagnosis and the detection of distant metastases can be prolonged, with an average latency period of approximately three years [4,5].

Although the spleen is involved in up to 30% of melanoma cases at autopsy [6], clinically apparent isolated metastases to this organ are exceedingly uncommon. Only a limited number of such cases have been documented in the literature [7,8]. In this report, we present a rare instance of solitary splenic metastasis from cutaneous melanoma, diagnosed three years following the initial curative treatment. We describe the patient's clinical presentation, imaging findings, and histopathological features, and provide a brief literature overview to highlight the significance of this unusual metastatic pattern.

## Case presentation

A 62-year-old male presented for elective surgery due to a suspicious splenic lesion. His medical history included hypertension and type 2 diabetes mellitus. Three years earlier, he underwent excision of a pigmented cutaneous nodular lesion located in the right pectoral region under local anesthesia. Histopathological analysis revealed a superficial spreading melanoma, Clark Level III, Breslow II, with resection margins of 0.8 mm and no evidence of lymphovascular invasion, microsatellitosis, or mitotic activity. Following receipt of the pathology report, a second surgical procedure was performed, consisting of re-excision of the surgical scar and dissection of the right axillary region. Histopathological examination confirmed metastatic melanoma in one of eight axillary lymph nodes (1/8). Examination of the resection margins of the re-excised specimen showed no evidence of increased melanocytic activity.

**Figure 1:**
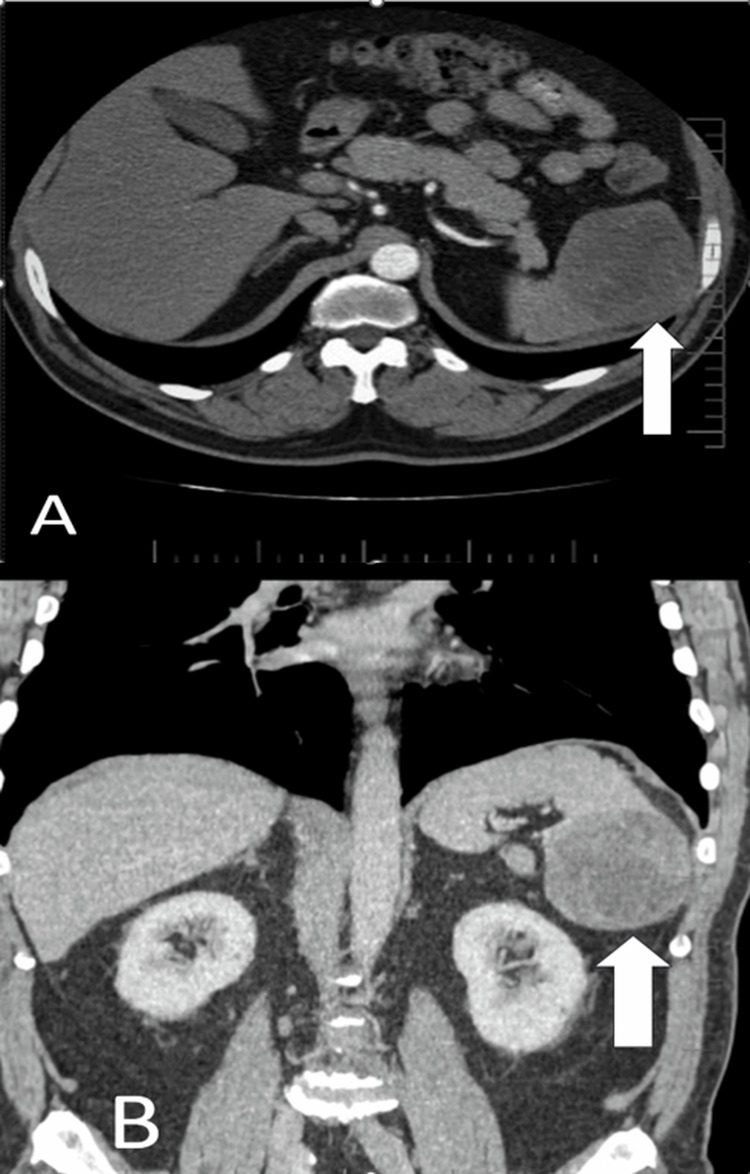
(A) Cross-section of abdominal organs: CT scan showing an oval, well-defined, hypodense lesion (~7 mm) in the lower pole of the spleen (arrow), with irregular peripheral soft tissue thickening and mild contrast enhancement. (B) Longitudinal section of abdominal organs: CT scan showing an oval, well-defined, hypodense lesion (~7 mm) in the lower pole of the spleen (arrow), with irregular peripheral soft tissue thickening and mild contrast enhancement.

On examination, physical findings and laboratory results were unremarkable. Follow-up imaging included whole-body CT scans performed every six months. PET/CT was not performed at that time. Abdominal ultrasound revealed an oval, solid lesion with mixed echogenicity in the lower pole of the spleen. CT imaging showed an oval, well-circumscribed lesion (~7 mm) with low attenuation (20-27 HU) and no enhancement after contrast administration. The lesion had irregular, thickened peripheral soft tissue zones (5-20 mm), with mild contrast enhancement (Figure 1).

Due to the uncertainty of imaging findings and the patient’s oncologic history, an open splenectomy was performed. Special preoperative measures were taken in preparation for the planned splenectomy, including vaccination against encapsulated bacteria (*Streptococcus pneumoniae*, *Haemophilus influenzae* type b, and *Neisseria meningitidis*), and optimization of hematologic and coagulation parameters. The postoperative course was uneventful.

**Figure 2:**
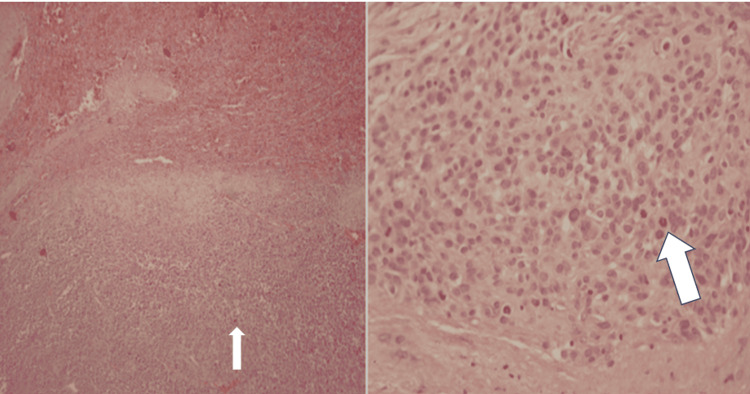
Histological section showing large, pleomorphic tumor cells, some epithelioid and others spindle-shaped, with vesicular nuclei, visible nucleoli, and high mitotic activity (arrows).

Pathologic examination showed a nodular tumor well-demarcated from normal splenic tissue. Microscopically, the tumor consisted of large pleomorphic cells with epithelioid and spindle morphology, vesicular nuclei, prominent nucleoli, and numerous mitotic figures (Figure 2).

Immunohistochemistry revealed the tumor cells were negative for leukocyte common antigen (LCA), broad-spectrum keratin, CD138, EMA, and Melan-A. They were diffusely positive for vimentin and S-100, and focally positive for HMB-45.

The final diagnosis was a secondary metastatic deposit of amelanotic melanoma (ICD-O histology code M8770/6). Genetic testing for common mutations, including BRAF and MEK, was not performed. Follow-up imaging, including whole-body CT scans performed every six months, showed no evidence of further metastases. Following surgery, the patient was referred to the Oncology Department, where a taxane-based adjuvant chemotherapy regimen was prescribed according to protocol. Regular follow-up included clinical examinations every six months, whole-body CT scans, and laboratory assessment of the S100 marker. PET/CT imaging and genetic testing for common mutations, including BRAF and MEK, were not performed due to financial and insurance limitations at the time. However, one year later, follow-up imaging revealed multiple metastases, and the patient died one year after splenectomy. 

## Discussion

Malignant melanoma arises from melanocytes and is predominantly located in the skin or mucosal surfaces [9]. The initial spread of this malignancy typically involves the skin (38%), lungs (36%), liver (20%), and brain [10]. Metastatic involvement of the spleen is unusual and may manifest several years - commonly between two and three - following the diagnosis of the primary lesion [11-13]. This infrequency may be attributed to anatomical and physiological characteristics of the spleen, such as its tortuous arterial supply, specific blood flow dynamics, absence of afferent lymphatic vessels, a robust capsule, and contractile properties that potentially inhibit tumor cell implantation [11,12]. When splenic involvement occurs, it usually signifies widespread metastatic disease, although most such lesions are clinically silent and discovered incidentally [11]. Solitary splenic metastases, as seen in our case, are particularly uncommon [12].

Radiologically, metastatic lesions of the spleen are frequently solitary and display hypoechoic features on ultrasound and hypodense characteristics on CT, sometimes with heterogeneous contrast enhancement if necrotic components are present [10]. Contrast-enhanced CT is considered a reliable modality for identifying and characterizing focal splenic lesions [14]. Nonetheless, due to overlapping imaging features, a multimodal diagnostic approach is often necessary to differentiate benign from malignant entities [15]. Contrast-enhanced ultrasound (CEUS) is a valuable tool in this context, although distinguishing metastases from lymphomas can be challenging; the presence of necrosis tends to favor metastasis [16]. In cases of suspected metastatic melanoma, confirmation via fine needle aspiration (FNA), core biopsy, or surgical sampling is recommended - except in brain metastases, where histologic confirmation is not always required prior to treatment [17]. In our patient, a biopsy of the splenic lesion could not be performed due to institutional limitations, including the absence of trained specialists for ultrasound-guided tumor biopsies. As systemic disease had not yet been confirmed at that time, surgical resection was pursued as a valid approach for managing isolated splenic involvement.

For patients diagnosed with Stage IV melanoma, therapeutic strategies include surgery, systemic immunotherapy, targeted therapy, chemotherapy, radiotherapy, and enrollment in clinical trials. Innovations in the molecular understanding of melanoma have significantly improved outcomes, particularly through immune checkpoint inhibitors and targeted agents, either as monotherapy or in combination [18]. Surgical intervention still holds a critical role, particularly when the disease is limited and resectable, offering the potential for durable responses [19]. Complete surgical resection in patients with solitary metastasis appears to offer the best chance of long-term survival [20].

## Conclusions

Solitary splenic metastasis from malignant melanoma is extremely rare. In patients with a history of melanoma, a solitary splenic lesion should raise suspicion for metastatic disease. Surgical resection remains the most effective treatment, offering the best chance for long-term survival.
